# Age and growth of stocked juvenile Shoal Bass in a tailwater: Environmental variation and accuracy of daily age estimates

**DOI:** 10.1371/journal.pone.0224018

**Published:** 2019-10-23

**Authors:** James M. Long, Michael J. Porta

**Affiliations:** 1 U.S. Geological Survey, Oklahoma Cooperative Fish and Wildlife Research Unit, Department of Natural Resource Ecology and Management, Oklahoma State University, Stillwater, Oklahoma, United States of America; 2 Department of Natural Resource Ecology and Management, Oklahoma State University, Stillwater, Oklahoma, United States of America; National Oceanic and Atmospheric Administration, UNITED STATES

## Abstract

Otolith microanalysis is often used to assess population age structure and growth of fishes during their early stages. Shoal Bass *Micropterus cataractae* is a recently described species of conservation concern and little is known regarding factors affecting their recruitment. In 2004, Georgia Department of Natural Resources (GADNR) and the US National Park Service (NPS) stocked Shoal Bass marked with oxytetracycline (OTC) in the Chattahoochee River near Atlanta, Georgia in an effort to restore this population, creating known-age fish to examine the effect of environment on daily age accuracy. We obtained samples of stocked juvenile (<150 mm) Shoal Bass from standard monitoring that occurred approximately 30–60 days after stocking in the Chattahoochee River to 1) validate daily rings for estimating age, hatch dates, and growth rates for stocked age-0 Shoal Bass, and 2) evaluate the effect of habitat (location) on age bias. Shoal Bass otoliths were examined for OTC marks and daily rings were counted in reference to OTC marks to assess age estimation accuracy. Age estimation accuracy ranged from -2 to -25 days and was influenced by the environment where Shoal Bass were captured, with greater inaccuracy in colder water temperatures. Fish collected from locations with colder temperatures displayed closer spacing of daily rings, potentially leading to greater underestimation of age. Growth rates of stocked Shoal Bass, corrected for age estimation error, ranged from 0.5 mm/day to 0.8 mm/day. This study demonstrates the effect of environmental variability on age inaccuracy and subsequent interpretation of results. Incorporating methods to assess age estimation accuracy is needed to understand interspecific differences in recruitment among black bass species in the variety of natural and human-modified environments they inhabit.

## Introduction

Otolith microanalysis is an often-used method to investigate population dynamics and age structure of black bass *Micropterus* species particularly during their early-life history. Since rings have been validated to form daily in otoliths of *Micropterus* species [1–5], researchers have used that information to understand early-life history based on ring counts. For instance, counts of daily rings in otoliths of Largemouth Bass *M*. *salmoides* in Alabama pond environments (≤ 2 ha) indicated that hatch date affected first-year survival [6]. Those that hatched earlier grew to larger sizes, switched to piscivory sooner, and thus gained more lipid reserves to survive their first winter than those hatched later. Wild Largemouth Bass in Lake Shelbyville, Illinois had the same pattern as those in Alabama ponds [7], suggesting hatch date generally affects recruitment of this species. In addition, early-hatch dates in Largemouth Bass have been shown to correlate with parent size in reservoirs, suggesting that larger adults spawned earlier, thus allowing their progeny to gain a larger size and switch to piscivory sooner than later-hatched fish born to smaller parents [8]. These studies have led to a general conceptual model for Largemouth Bass where hatch time affects ontogeny of piscivory and the survival through the summer and winter to recruit to age-1 [9], although this may vary with latitude [10]. However, as these studies also demonstrate, most age-based metrics of recruitment have been studied for Largemouth Bass in lentic systems (even validation studies), leaving a gap in our understanding in other related species and lotic environments.

Whether recruitment in *Micropterus* species is a function of environment (lentic v lotic) or species-specific differences is unclear. For instance, riverine Smallmouth Bass *M*. *dolomieu* in the North Anna River, Virginia showed correlations between swim-up date and growth rate counter to that generalized for Largemouth Bass in lentic habitats [11]. In the river, Smallmouth Bass that hatched early in the year grew slowly whereas late-hatched individuals grew rapidly, eventually becoming the same size by the middle of summer [11]. But, in similar environments, recruitment of Largemouth Bass and Spotted Bass *M*. *punctulatus* showed differential responses to hydrology over a 5-year period in Normandy Reservoir, Tennessee [12]. Hampering our ability to discern species-specific differences is the lack of validation studies for most *Micropterus* species. To date, daily rings have been validated in otoliths of Largemouth Bass [1–2,4], Smallmouth Bass [3,13], and Spotted Bass [5], but not for other congeners.

Little is known about Shoal Bass *M*. *cataractae* recruitment, but this species is generally described as a fluvial-specialist, intolerant to reservoirs [14–15]. Relative to understanding early life history through otolith microstructure, it is important to be able to accurately count and identify daily rings, which has not been corroborated for Shoal Bass. Rings in otoliths from Shoal Bass raised in a Georgia hatchery were concluded to have formed daily [16]. However, relying on studies from relatively stable hatchery systems, such as is often the case for most daily age validation studies, may result in process errors [17] where formation of rings can vary among structures or systems. Daily ring formation is affected by both obligatory and facultative processes, with temperature regime (mean and variation) being highly influential on the ability to accurately count [18–20]. Without additional data on the ability to accurately count daily rings in Shoal Bass under conditions in the wild, it is unclear how hatch date phenology might affect recruitment. Shoal Bass appear to have a contracted hatching period, lasting approximately 12 days in a Georgia hatchery [16], and most (73–93%) spawning was observed to occur over a 11-d to 18-d period in the Chipola River, Florida [21], suggesting low potential for phenological effects to manifest.

In 2004, the Georgia Department of Natural Resources (GADNR) and the US National Park Service (NPS) conducted restoration stocking of Shoal Bass in the Chattahoochee River near Atlanta, Georgia, providing an opportunity to determine daily age estimation accuracy and infer environmental effects. The stocking protocol involved rearing fish spawned in hatchery ponds, marking stocked fish with oxytetracycline (OTC), and stocking at multiple sites along an environmental gradient. The 2004 year-class was one of five that were stocked in the Chattahoochee River and the most successful [22], making it particularly useful to study at an early-life stage. As a result, we had a unique opportunity to not only investigate how these stocked fish responded to a natural environment through otolith microanalysis, but to assess the accuracy of those analyses in relation to dates associated with spawning in the ponds and OTC marks. The objectives of this study were to 1) validate daily rings for estimating age, hatch dates, and growth rates for stocked age-0 Shoal Bass, and 2) evaluate the effect of habitat (location) on age bias.

## Study site

**Fig 1:**
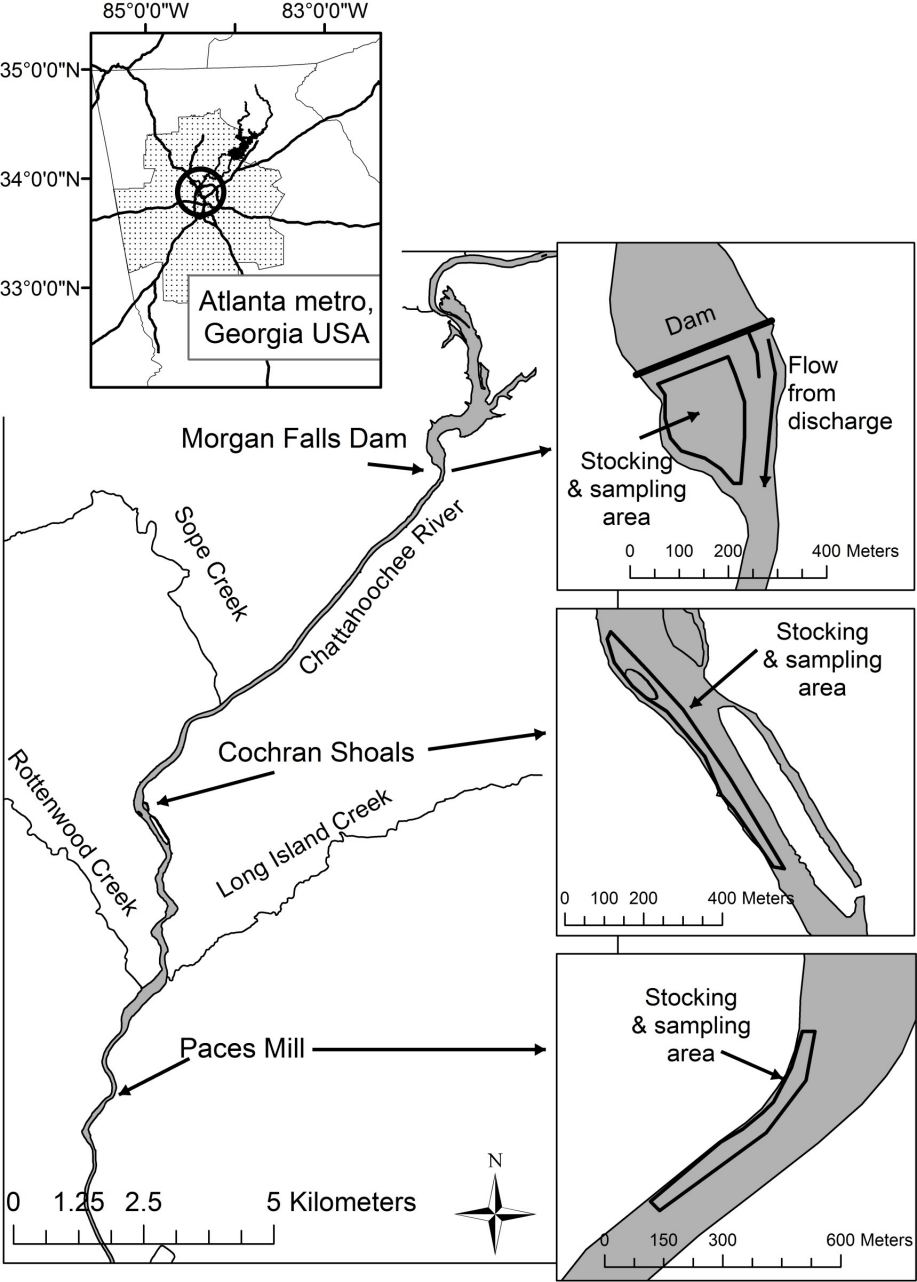
Map of the upper Chattahoochee River below Morgan Falls Dam. Juvenile Shoal Bass were stocked at three sites in the river in May and June 2004. Juvenile Shoal Bass were collected at these three sites in June and August 2004.

The study area (33.924976 latitude, -84.429949 longitude) is located in the NPS Chattahoochee River National Recreation Area (CRNRA) in the Atlanta, Georgia metropolitan area. The CRNRA manages several land units bordering the Chattahoochee River downstream of Morgan Falls Dam, including Cochran Shoals and Paces Mill (Fig 1). The Chattahoochee River below Morgan Falls Dam is unnaturally cold due to the release of hypolimnetic water from upstream Buford Dam, but exhibits a longitudinal gradient in water temperature, being colder immediately downstream of Morgan Falls Dam and warming through the downstream reaches at Cochran Shoals and Paces Mill [22–23]. Despite this warming trend in the river, the water temperature regime in this reach of river remains marginal for warmwater black basses.

## Methods

In 2004, approximately 40,000 Shoal Bass were spawned and reared in 0.4 ha earthen hatchery ponds at the Steve Cocke Fish Hatchery in Dawson, Georgia (approximately 240 km due south of Atlanta), which is operated by the Georgia Department of Natural Resources. On May 4, all Shoal Bass (~25 mm TL) were marked with oxytetracycline (OTC); 30,000 were stocked in the river, equitably among three study sites (below Morgan Falls Dam, Cochran Shoals, and Paces Mill; Fig 1) and the remaining 10,000 fish were put back in the hatchery pond to be reared for another 30 days. On June 9, the hatchery pond was harvested; the Shoal Bass (~60 mm TL) were given a second OTC mark, and stocked in the river equitably among the three sites. Marking and stocking of fish was carried out by Georgia Department of Natural Resources.

**Fig 2:**
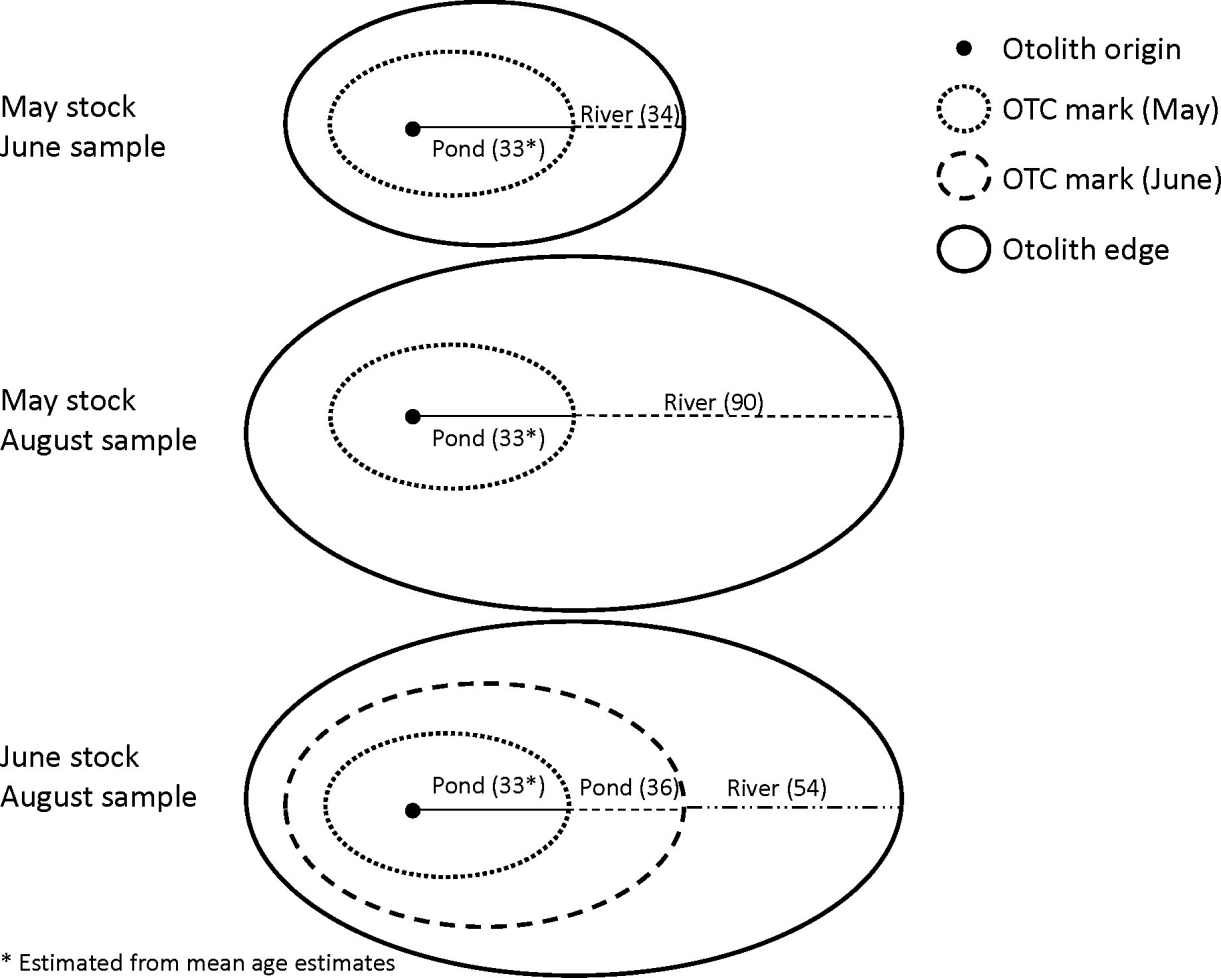
Illustration of oxytetracyline (OTC) marks and the known age (days) associated with each. For fish stocked in May and sampled in June, 33 days in the pond (estimated as the mean daily age from counts of rings in otoliths) and 34 days in the river elapsed. For fish stocked in May and sampled in August, 33 days in the pond and 90 days in the river elapsed. For fish stocked in June and sampled in August, 33 days in the pond elapsed in the pond until the first OTC marking episode, and another 36 days elapsed in the pond until the second OTC marking episode; 54 days elapsed between the time the fish were stocked in the river and subsequently sampled.

Fish used for this study came from samples of stocked juvenile (<150 mm) Shoal Bass as part of routine post-stocking monitoring that was undertaken in 2004 by personnel with Georgia Department of Natural Resources and US National Park Service at the three stocking sites in the Chattahoochee River (below Morgan Falls Dam, Cochran Shoals, and Paces Mill; Fig 1). Fish were obtained by use of backpack electrofishing on June 7 (34-d post May stocking) and August 2 (54-d post June stocking and 90-d post May stocking; Fig 2). Specimens provided to the authors for analysis in 2009 were stored in 70% ethanol and each individual was measured for total length (TL; mm) prior to dissection under the auspices of Oklahoma State University’s Institutional Animal Care and Use Committee protocol AG-09-20. Otoliths (sagittae) were removed from each fish, mounted concave side up with thermoplastic cement on individually numbered glass slides [1], and polished with 600-grit wet/dry sandpaper until a flat plane from the center to the edge was obtained and the inner-most rings were clearly discernable. Otoliths were viewed with a drop of mineral oil (as a clarifying medium) under a compound microscope (40-100x). We inspected for the presence and location of OTC marks to determine the stocked cohort (1 for May-stocked fish and 2 for June-stocked fish), and we counted rings from the nucleus to the OTC mark and from the OTC mark to edge. For June-stocked fish, rings were also counted from the 1^st^ mark to the 2^nd^ mark. Total age for each Shoal Bass was the sum of ages between nucleus, OTC marks, and the edge. Each otolith was read independently in random order three times by a single individual and the average of these three counts (rounded up to nearest whole number) was used for the age estimates [5].

We assessed accuracy for stocked Shoal Bass by calculating the absolute difference between known-age and estimated age and used a generalized linear mixed model with a lognormal distribution (Proc GLIMMIX, solution link = log dist = logn option; SAS Institute) to determine statistical differences among stocking-sampling cohorts (3 groups illustrated in Fig 2) and environment (pond and three river locations; Fig 1). We considered environment a random effect and accounted for individual variation in fish otoliths within the environment and to properly account for multiple otolith measurements within each fish otolith (e.g., age error in pond before stocking and age error in river after stocking that are both observable in the same, one otolith) using a random statement and subject = “fish ID” option. We used LSMEANS with PDIFF option as a post-hoc comparison to determine where significant differences occurred when the overall model was significant. From the post-hoc analyses, we considered four comparisons to determine for significant differences in age estimation: 1) among pond environments (pond-pond), 2) between pond and river environments within each stock-sample cohort (pond-river within cohorts), 3) within river-site locations among stock-sample cohorts (within-river among cohorts), and 4) within stock-sample cohorts among river site locations (within cohort among river sites). Known-age for the May-stocked fish was considered the mean estimated age from the nucleus to the first OTC mark; known-age for the remaining groups was the elapsed days between OTC marks and the otolith edge at the time of sampling. At each stocking location in the river, we used estimated ages to back-calculate hatch dates (sample date–age) and estimate growth rate (TL at sample date / age). Using age-estimation error from OTC marks in Shoal Bass otoliths, we corrected age estimates for Shoal Bass and calculated corrected hatch dates and growth rates. We also constructed histograms of hatch date frequencies using raw (uncorrected) and corrected age data to determine potential effects of age error on interpretation of hatch phenology in the wild.

To determine the effect of environment on growth, we measured the distance associated with ten daily increments before and after OTC marks and compared among stocking sites and cohorts. For this analysis, we used fish captured in June that had one OTC mark and fish captured in August that had two OTC marks. We divided the post-OTC distance by the pre-OTC distance to determine the proportional growth between stocking environments (i.e., 1.0 would indicate no effect of environment, <1.0 would indicate reduced growth after stocking, and >1.0 would indicate increased growth after stocking). We tested for differences in proportional growth among stocking sites (below Morgan Falls Dam, Cochran Shoals, and Paces Mill) and stock-sample cohorts with two-way ANOVA (Proc GLM, SAS Institute). Proportional growth for the May stocked fish sampled in June would indicate the effect of the river environment. Proportional growth for the June stocked fish would indicate the effect of remaining in the pond at the first OTC mark when the May stocked fish were sampled (pond effect) and indicate the effect of being stocked in the river at the June date at the second OTC mark (river effect) after being sampled in August. Thus, this analysis resulted in two separate measures of the effect of being stocked in the river and one measure of the effect of remaining in the pond.

To help interpret our age error results, we plotted available water temperature data in relation to stocking and sampling dates. Water temperature is a significant factor affecting growth of fish (and interpretation of daily rings in otoliths), but other factors such as habitat and prey availability can also contribute. In this section of the Chattahoochee River, temperature exhibits a longitudinal trend, along with measures of species diversity (e.g., prey diversity) and diversity of habitat [23], but temperature was the only variable that was readily accessible for comparison with our results. Daily water temperature (minimum and maximum) for the Chattahoochee River was obtained from the two U.S. Geological Survey (USGS) stream monitoring gages in the study area (https://waterdata.usgs.gov/ga/nwis/current/?type=flow&group_key=basin_cd): upstream station 02335810 at Morgan Falls Dam and downstream station 02336000 at Atlanta, Georgia (~1.6 km from Paces Mill). Opportunistic point samples of water temperature were obtained from GADNR for the hatchery pond where Shoal Bass were reared (GADNR, unpublished data) and from GA Environmental Protection Division (EPD; http://www1.gadnr.org/dnr/wrdb/homePage.do) station RV_12_3870 between the Morgan Falls Dam and the Cochran Shoals stocking locations in the Chattahoochee River.

## Results

**Fig 3:**
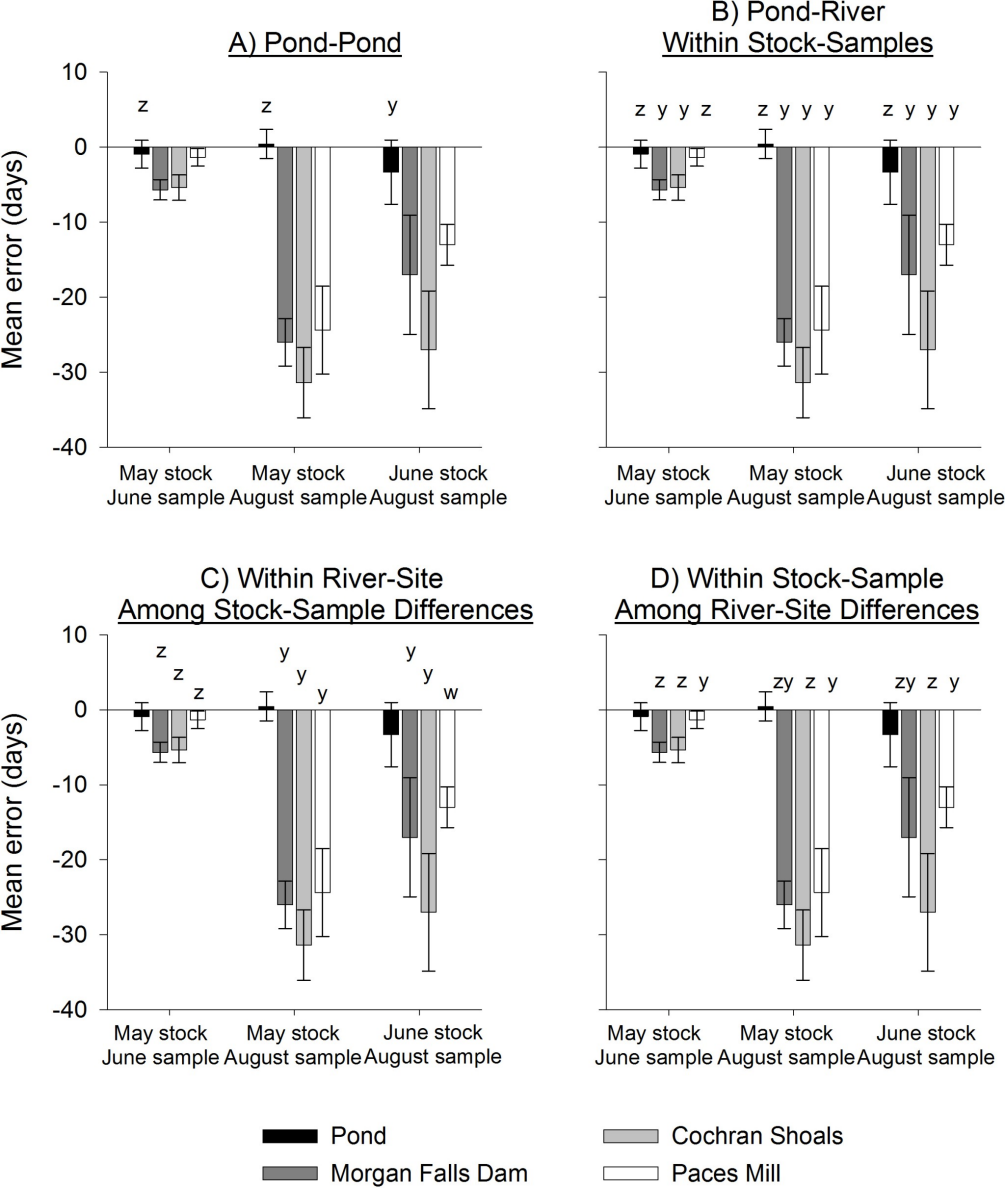
Mean age error (days; ± 1 SD) of juvenile Shoal Bass in different environments. The amount of age estimation error was tested among sets of environments according to time of stocking, oxytetracyline (OTC) marking (May or June) and time of sampling (June or August) in 2004. Four comparisons from a generalized linear mixed model were made: (A) pond environment among stock-sample combinations, (B) pond compared to sites in river within each stock-sample combination, (C) among stock-sample combinations for each of the three river sites, and (D) among each of the three river sites for each stock-sample combination. Dissimilar letters above the bars indicate significant differences at *P* ≤ 0.05. See Fig 2 for a diagrammatic representation of stocking, OTC marking, and sampling scheme for these comparisons.

We captured and examined otoliths of 177 Shoal Bass from both sampling events (Table 1). Age error resulted in underestimates of age, which was evident from OTC-marked Shoal Bass otoliths and was dependent on environment and stocking location (Proc GLIMMIX, environment*cohort interaction F_6,17_ = 29.43, *P* < 0.01; Table 2; Fig 3). Among residency times in the pond, mean error was greatest when Shoal Bass were oldest in the pond during June (June stock, August sample), compared to being reared in the pond from hatch until being stocked in May (Fig 3A). When compared to the pond environment, age error was greatest for each of the stocking locations in the river, except for the furthest downstream site (Paces Mill) at the earliest stocking-sampling time where age error was not significantly different from that observed in the pond (May stock, June sample; Fig 3B). Within river sites, age error was always greatest for older fish at Morgan Falls Dam and Cochran Shoals (within river-site, among stock-sample cohorts; Fig 3C). For Shoal Bass at Paces Mill (farthest downstream site), age error differed among all cohorts, in concert with age and residency time in the pond. For example, the May-stocked cohort in June was 67 days old, spending 33 days in the hatchery pond and 34 in the river and cumulative mean age error was approximately 2 days. The same stocking-cohort sampled in August was 123 day old, having spent 90 days in the river and had cumulative mean underestimates of age of approximately 25 days. Conversely, the June stocked cohort sampled in August was also 123 days old, but only spent 54 days in the river (about half the time as the May-stocked cohort) and had cumulative underestimates of age of only 18 days. Among river locations, age error was least for fish sampled in June at Paces Mill (the most downstream site), and equal for fish at Morgan Falls and Cochran Shoals (Fig 3D). For August samples, age error was least at Paces Mill compared to Cochran Shoals, but similar compared to those from Morgan Falls Dam.

**Table 1:** Age-based metrics of Shoal Bass reared in hatchery ponds and stocked at three sites in the Chattahoochee River, Georgia in 2004.

River site	*N*	Mean TL (mm)	Estimated age (days)	Uncorrected hatch date	Uncorrected mean growth rate (mm/day)	Cumulative error (days)	Corrected age (days)	Corrected hatch date	Corrected mean growth rate (mm/day)
**6/7/2004 (May stock, June sample)**									
MFD	21	44.2	61.9	4/6/2004	0.7	-5.1	67	4/1/2004	0.7
CS	21	52.8	61.7	4/6/2004	0.9	-5.3	67	4/1/2004	0.8
PM	60	53.3	64.7	4/3/2004	0.8	-2.3	67	4/1/2004	0.8
**8/2/2004 (May stock, August sample)**									
MFD	8	55.9	97.5	4/26/2004	0.6	-25.5	123	4/1/2004	0.5
CS	21	72.9	91.7	5/2/2004	0.8	-31.3	123	4/1/2004	0.6
PM	29	76.0	97.6	4/26/2004	0.8	-25.4	123	4/1/2004	0.6
**8/2/2004 (June stock, August sample)**									
MFD	3	85.7	102.9	4/21/2004	0.8	-20.1	123	4/1/2004	0.7
CS	6	83.0	91.6	5/2/2004	0.9	-31.4	123	4/1/2004	0.7
PM	8	92.5	105.0	4/18/2004	0.9	-18.0	123	4/1/2004	0.8

TL, total length, MFD, Morgan Falls Dam, CS, Cochran Shoals, and PM, Paces Mill.

Fish were reared to 33 days old, marked with oxytetracycline (OTC, 1^st^ mark), stocked in the river May 4, and sampled June 7. A portion of fish was reared for an additional 36 days size to a larger size at the hatchery before being marked again with OTC (2^nd^ mark), stocked in the river June 9, and sampled August 2. See Fig 1 for location of sites in the river and Fig 2 for illustration of OTC marks on otoliths.

**Table 2:** Mean age estimates of Shoal Bass reared in hatchery ponds and stocked at three sites in the Chattahoochee River, Georgia in 2004.

Site	Count of rings to 1st mark (May stock, June and August sample)	Count of rings after 1st mark (May stock, June sample)	Count of rings after 1st mark (May stock, August sample)	Count of rings between 1st and 2nd mark (June stock, August sample)	Count of rings to 2nd mark (June stock, August sample)	Count of rings after 2nd mark (June stock, August sample)
**Known age**	33*	**34**	**90**	**36**	**69ˠ**	**54**
Morgan Falls Dam	33.5 (0.5, 29)	28.3 (-5.7, 21)	64 (-26.0, 8)	32.3 (-3.7, 3)	69.3 (2.3, 3)	37 (-17.0, 3)
Cochran Shoals	33.1 (0.1, 42)	28.6 (-5.4, 21)	58.6 (-31.4, 21)	31.5 (-4.5, 6)	63.2 (-3.8, 6)	27 (-27.0, 6)
Paces Mill	32 (-1.0, 89)	32.7 (-1.4, 60)	65.6 (-24.4, 29)	32 (-4.0, 8)	67.9 (0.9, 8)	41 (-13.0, 8)

Measurement error in days and the sample sizes are listed in parentheses.

Fish were reared to 33 days old, marked with oxytetracycline (OTC, 1^st^ mark), stocked in the river May 4, and sampled June 7. A portion of fish was reared for an additional 36 days size to a larger size at the hatchery before being marked again with OTC (2^nd^ mark), stocked in the river June 9, and sampled August 2. See Figs 2 and 3 for illustration.

* estimated as mean among sites.

**ˠ** calculated as 33 days mean age to 1^st^ mark + 36 known days between 1^st^ and 2^nd^ marks

**Fig 4:**
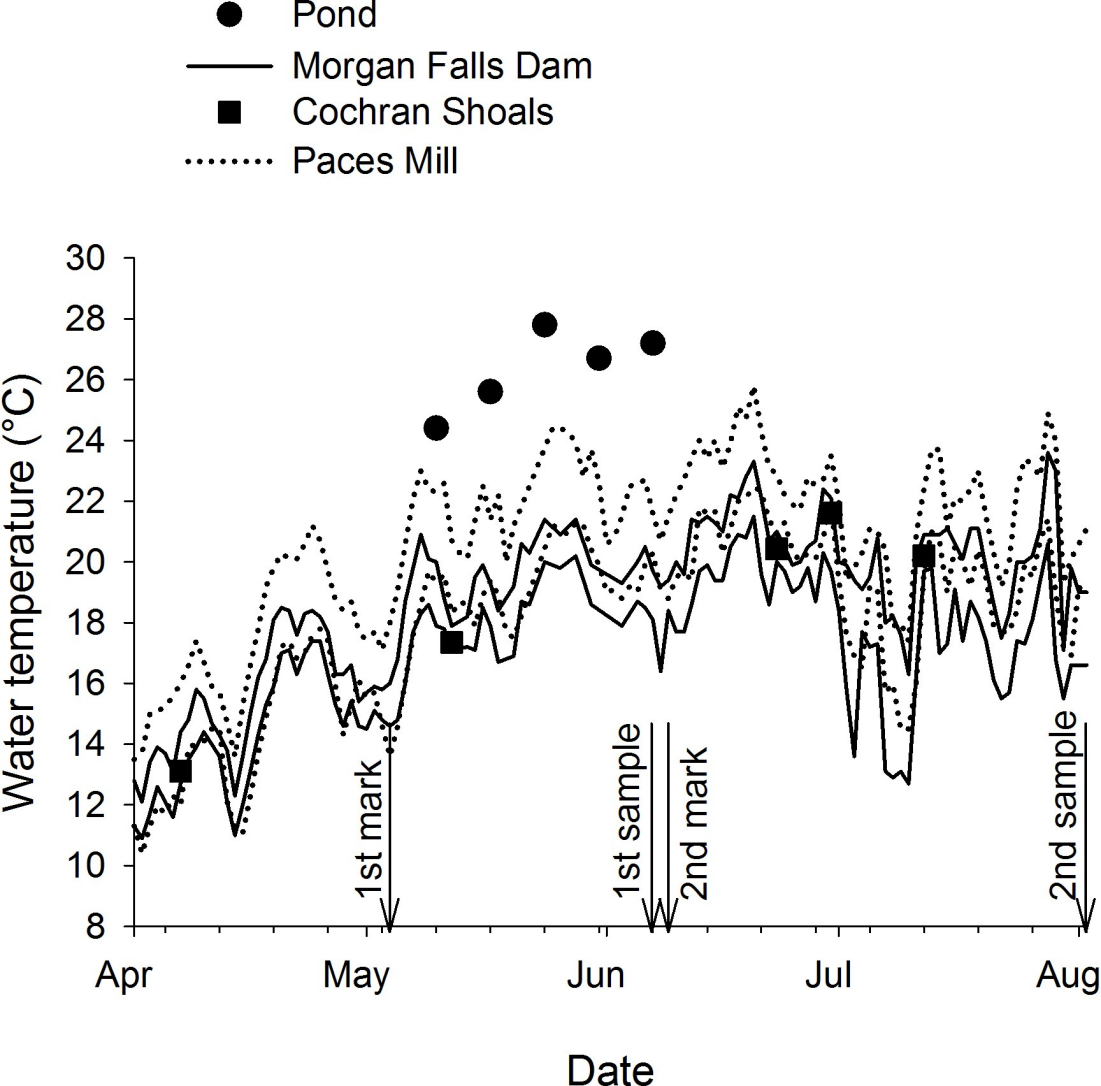
Temperature in the Chattahoochee River and the hatchery pond where Shoal Bass were sampled. The paired lines represent the maximum and minimum daily temperatures for Morgan Falls Dam and Paces Mill in the Chattahoochee River. Squares indicate point temperature values for the Chattahoochee River in the vicinity of Cochran Shoals. Circles indicate the hatchery pond water temperature that was taken haphazardly during the time Shoal Bass were present. The dates on which Shoal Bass were marked and sampled are indicated with arrows. See Fig 1 for locations in the river.

We measured increment widths for 101 Shoal Bass captured in June that had 1 OTC mark and 17 captured in August that had 2 OTC marks (the remainder of fish captured in August only had 1 OTC mark), allowing for analysis of growth effects (Table 3). Proportional change in distance of 10 increments before and after OTC marks suggested that growth of stocked Shoal Bass was reduced when stocked in the river regardless of stocking cohort and river site (2-way ANOVA, river-site*cohort interaction F_4,126_ = 1.02, *P* = 0.40). In June and August, the distance of 10 rings after stocking in the river was approximately one-half the distance of 10 rings before stocking whereas the distance was approximately equal for the cohort stocked back in the pond (2-way ANOVA, cohort F_2,126_ = 111.68, *P* < 0.01). Reductions in growth were also less for fish stocked at Paces Mill compared to those stocked further upstream (2-way ANOVA, river site F_2,126_ = 6.21, *P* < 0.01). Reductions in growth observed on the otoliths coincided with water temperature differences between the hatchery pond and the river sites (Fig 4). Opportunistic water temperature samples from the pond exceed the maximum temperatures observed in the river by 2° to 7° C. Water temperatures in the river increased downstream, being warmest at the most downstream site (Paces Mill). Furthermore, mean daily water temperature fluctuation (maximum–minimum) was greatest downstream (2.8° C at Paces Mill) compared to upstream (1.9° C at Morgan Falls Dam).

**Table 3:** Mean proportional distance of 10 daily rings post-OTC mark compared to 10 daily rings pre-OTC mark for Shoal Bass (*N*) reared in hatchery ponds and stocked at three sites in the Chattahoochee River, Georgia.

Site	River effect 1st mark (May stock, June sample)	Pond effect 1st mark (June stock, August sample)	River effect 2nd mark (June stock, August sample)	Site mean
Morgan Falls Dam	0.56 (21)	1.07 (3)	0.53 (3)	0.72 ^z^ (27)
Cochran Shoals	0.55 (20)	1.01 (6)	0.52 (6)	0.69 ^z^ (32)
Paces Mill	0.63 (60)	1.06 (8)	0.70 (8)	0.79 ^y^ (76)
Cohort mean	0.58^z^ (101)	1.05^y^ (17)	0.58^z^ (17)	

Fish were reared to 33 days old, marked with oxytetracycline (OTC, 1^st^ mark), stocked in the river May 4 (1^st^ stocking), and sampled June 7 (1^st^ sample). A portion of fish was reared for an additional 36 days size to a larger size at the hatchery before being marked again with OTC (2^nd^ mark), stocked in the river June 9 (2^nd^ stocking), and sampled August 2 (2^nd^ sample). See Fig 2 for illustration. Significant differences among sites (last column) and cohorts (last row) are indicated with superscript letters.

**Fig 5:**
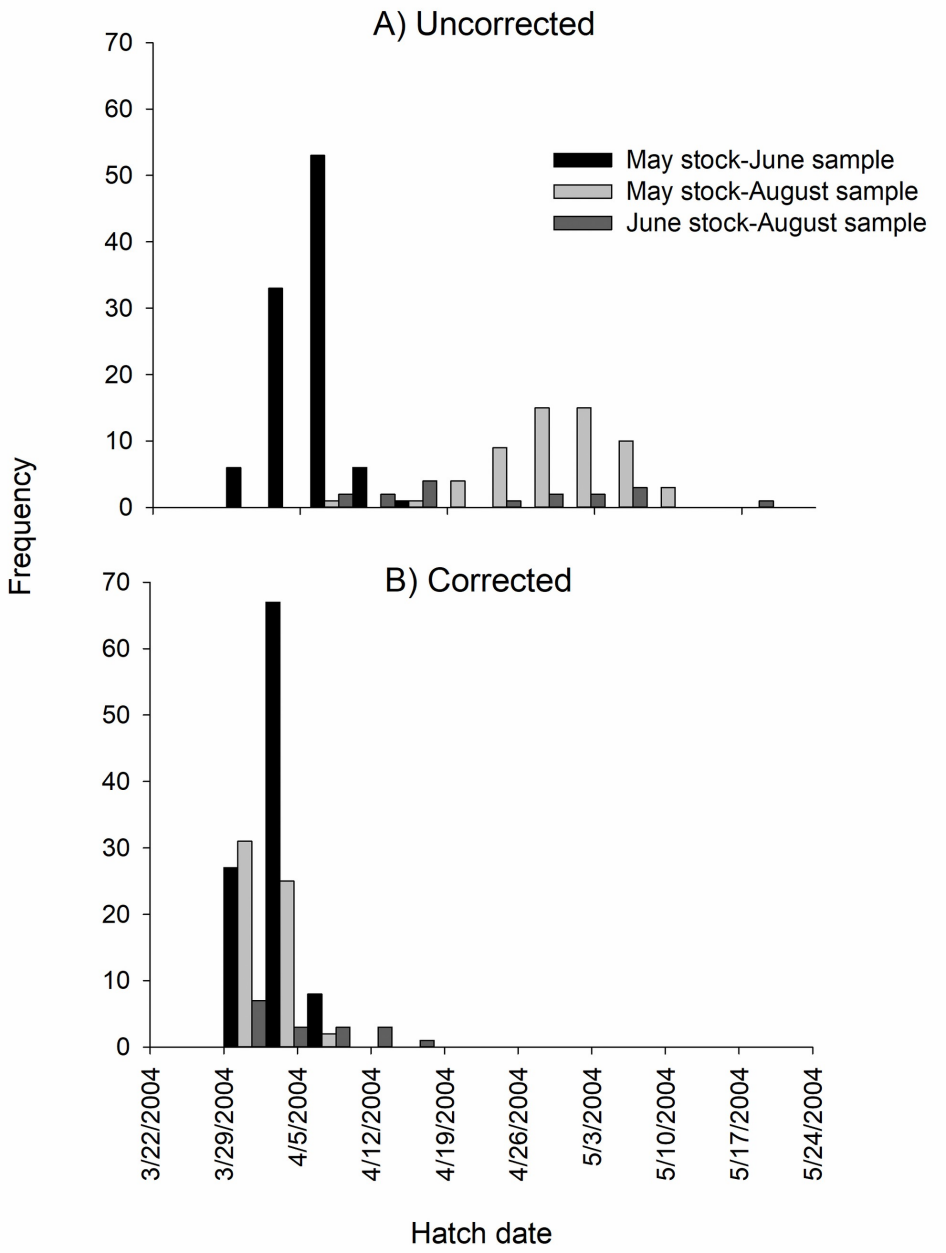
Back-calculated hatch dates of Shoal Bass. (A) Hatch dates were estimated using raw daily age counts (uncorrected) and (B) adjusted for age estimation bias (corrected).

Using age-estimation error to correct back-calculated hatch dates and growth rates produced varied results depending on sample dates. For Shoal Bass, where age error was quantifiable because of OTC marks, hatch dates were corrected by about one week for fish sampled in June compared to almost a month correction for fish sampled in August (Table 1). Histograms of uncorrected hatch dates were late March-early April for fish captured in June, but late April to early May for fish captured in August, suggesting a bi-modal distribution of hatching (Fig 5). However, when corrected for age error, hatch dates occurred from late March to early April regardless of sample date, suggesting unimodal hatching. Corrected growth rates were adjusted by 0.1 to 0.2 mm/day, ranging from 0.5 mm/day to 0.8 mm/day.

## Discussion

In this study, we observed a direct effect of environment on daily age accuracy, which has implications for similar studies in variable ecosystems. Because fish growth is tightly coupled with temperature [24], we believe water temperature is the main driver of our results, although the role of mean or range (i.e., fluctuation) is still unresolved. Of studies that have examined the role of temperature fluctuations on accuracy of daily ring counts in otoliths, the results have generally found that fluctuating temperatures improve daily ring clarity and accuracy. In Chinese Sucker *Myxocyprinus asiaticus*, daily increments were reported to be clear and easily distinguishable from fish held in fluctuating water temperatures (18–25°C) and formed 11 increments over 11 days compared to those held at constant 24°C [19] that formed 5 increments over 11 days. Similarly, Colorado Pikeminnow *Pytchocheilus lucius* age estimates were affected by temperature regimes [25]. Although slope estimates between known and otolith-estimated age were not significantly different from zero for any of the temperature regime experiments over a 165 day period, 95% prediction limits of known age were more than twice as large for fish held at constant 22°C (±17.7 days) compared to fish held in fluctuating (±2.5°C) environments around 18, 22, and 26°C (±7.2 days). Alternatively, Smallmouth Bass held for 30 days post-hatch formed clearly discernable daily increments regardless of temperature regime (constant 20°C and ±2° around 20°C) [13]. Thus, constant temperature regimes appear to inhibit daily ring formation, but the effect of fluctuating temperatures still remains unresolved because these studies introduced a variable temperature regime similar to a diel period, but did not introduce varying fluctuations around a similar mean (e.g., ±2° around 20°C compared to ±4° around 20°C). But, as previously stated, the environments in our study differed in ways other than temperature (e.g., physical habitat and organism diversity [prey availability]) requiring additional study to understand the mechanism of the differences we observed. However, the natural conditions where our study occurred, with positively marked rings for accuracy, provide strong implication that our results could apply to a variety of situations.

Significant is the fact that we used artificially-reared fish that likely were larger than if they had been produced naturally in the wild [26]. For Shoal Bass, larger-stocked fish have been shown to survive better to adulthood than smaller-stocked fish [22]. Furthermore, it was clear that age accuracy was better, and consistent over time, when these fish were residing in the hatchery pond. Although, fish stocked in the most-downstream, warmest part of the river (Paces Mill) exhibited similar accuracy in June to the time when fish were in the pond, which demonstrates that natural environmental variables can affect daily ring accuracy and its resulting calculations of growth and hatch date estimates. Regardless, the role of fish growth on daily age accuracy was evident as ring spacing coincided with age accuracy, and was most comparable to the pond environment at the most downstream, warmest site in the river (Paces Mill).

Our results can help resolve an important paradigm of recruitment in black bass. For Largemouth Bass in lentic systems, early-hatched fish have been found to reach larger sizes sooner, switching to piscivory earlier, gaining a greater amount of lipids, and thus surviving their first winter better than later-hatched individuals [6,27–28]. In contrast, Smallmouth Bass in lotic systems have been found to have no advantage for hatching earlier because of temperature-mediated growth effects, with earlier-hatched individuals being born during cooler periods than later-hatched individuals and thus growing slower [29]. In this instance, both cohorts reach similar sizes as they approach their first winter negating any benefit to hatch date. Little information exists for understanding environmental effects on age-0 Shoal Bass, but river systems within their native range include spring-fed systems with stable temperatures (Chipola River, Florida [30]) to reservoir-regulated systems with variable temperature (Chattahoochee River, Alabama-Georgia [31]), Growth of other stream-dwelling species, such as Smallmouth Bass, have been affected by factors that regulate temperature, such as shade and groundwater influence [32], and our study has shown how growth can affect daily age interpretation making this a fruitful avenue for further study.

Although many hypotheses have been presented for explaining differences in early-life growth and hatching time (e.g., temperature regime, food quality, habitat variation, sampling biases) [6,28,31], none have suggested the effect of age inaccuracy, which we documented in this study as varying in concert with environmental conditions and can have a dramatic effect on interpretation of results. Importantly, these studies of recruitment have relied on daily age estimates, which are dependent on assumptions of accuracy. Generally, these studies assume that daily ages are accurate because their rings have been validated in a previous study [e.g., 1–2,13]. However, a method may be valid, but the results applied to a different setting may not necessarily be accurate [17]. Typically, aging studies at a particular location will cite a validation study conducted elsewhere and then use some measure of precision (e.g., multiple reads or readers) to assume accuracy can be transferred to the environment of interest. Few studies involving juvenile black bass have attempted stronger, *in-situ* corroboration of daily age estimates. For example, although consistently locating the 6, 12, 24, and 50 increments is meant to ensure accurate daily age estimates [11], this is still a measure of precision, not accuracy, because none of those increments were marked at a known age. Rather, the readers established their precision in their ability to repeatedly count to a set value. In our study, we had up to 2 OTC marks to use as reference, which served as a check on accuracy of age estimates.

Although we recognize the high level of added burden it would take to mark and later recapture sufficient individuals to assess aging accuracy of individuals in the environment of interest, we suggest this is the next logical step for others interested in the role of recruitment for black basses, especially in variable environments. Considering that temperature and food affect fish growth in addition to affecting formation and discernibility of daily rings in otoliths [18], assessing the accuracy of estimates obtained from otoliths as part of understanding the effect of environmental variation on fish growth and survival is going to be necessary. As the paradigm of conserving black bass diversity becomes more prevalent [33–34] in light of their recognized conservation needs [35], so too is the need to understand early life history, including the ability to accurately estimate daily age [36].
